# Proposal for a Disease Activity Score and Disease Damage Score for ADA2 Deficiency: the DADA2AI and DADA2DI

**DOI:** 10.1007/s10875-023-01638-w

**Published:** 2023-12-22

**Authors:** Giorgia Bucciol, Amanda K. Ombrello, Eugene P. Chambers, Isabelle Meyts

**Affiliations:** 1 Laboratory of Inborn Errors of Immunity, Department of Microbiology, Immunology and Transplantation, KU Leuven, Leuven, Belgium; 2 Childhood Immunology, Department of Pediatrics, Leuven University Hospitals, Herestraat 49, 3000 Leuven, Belgium; 3 National Human Genome Research Institute, National Institutes of Health, Bethesda, MD USA; 4 Vanderbilt University, Nashville, TN USA; 5 DADA2 Foundation, Nashville, TN USA

To the Editor,

Autosomal recessive deficiency of adenosine deaminase 2 (DADA2) was described in 2014 as the cause of an autoinflammatory syndrome characterized by vasculitis, manifesting mainly as polyarteritis nodosa, strokes, and livedo reticularis [1, 2]. It is caused by biallelic loss of function mutations in the ADA2 gene. Its prevalence has been estimated at 1:222,000, based on carrier frequency of the pathogenic genetic variants, with 35,000 affected individuals worldwide [3]. The originally described phenotype, which was centered on vasculitis and rheumatological manifestations, has expanded to include a spectrum of immunological and hematological manifestations, such as hypogammaglobulinemia, infections, cytopenia, bone marrow failure, and malignancies ([1, 2, 4] supplementary references E1-E8). A consensus statement on the diagnostics, follow-up, and treatment of patients with DADA2 has recently been published [5]. The mainstay of treatment is a disease-modifying therapeutic approach by tumor necrosis factor (TNF) blockade with etanercept or other TNF-inhibitors, which have been shown to control vasculitis and effectively prevent strokes [4, 5]. Antibody substitution and antibiotics are used to treat hypogammaglobulinemia and recurrent infections, while hematopoietic stem cell transplantation (HSCT) remains for now the only curative option in patients with severe cytopenia, deep immunodeficiency, or bone marrow failure ([4], supplementary E9,E10).

Various disease activity indexes are being used in the context of complex autoimmune or autoinflammatory conditions, such as juvenile idiopathic arthritis, childhood vasculitis, hereditary periodic fever syndromes, or systemic lupus erythematosus (SLE) (supplementary E11-E15). In the context of individually ultrarare inborn errors of immunity (IEIs), disease activity indexes have been shown to be useful for IEIs with many comorbidities, such as common variable immunodeficiency (CVID), or IEIs characterized by immune dysregulation and autoimmunity, exemplified by the development of the immune deficiency and dysregulation activity (IDDA) score to assess disease severity in lipopolysaccharide and beige-like anchor protein (LRBA) deficiency, which has then been extended to include other IEIs with immune dysregulation as the IDDA2.1 score (supplementary E16-E18).

Given the complex and often evolving phenotype encountered in DADA2, this condition would similarly benefit from the development of a disease severity score to aid in the longitudinal assessment of patients, intra- and inter-patient comparisons, and the evaluation of long-term outcomes with different types of therapy. Therefore, we here propose a disease activity index (DADA2AI) and a disease damage index (DADA2DI) that take into account the organ systems possibly affected in DADA2 according to the current knowledge of the disease as reported in large international patients’ cohorts ([4], supplementary E9,E10,E19,E20). The scoring system was developed thanks to the experience with other established disease activity indexes such as the IDDA, IDDA2.1, and SLE scores (supplementary E15,E17,E18). In the disease activity index, we attribute a score of 0 to 3 to 38 clinical/imaging items and of 0 to 4 to four laboratory values (Table 1 and DADA2AI score calculator in the supplement). The score considers not only the presence or absence of symptoms, but also their longitudinal evolution, as reflected by the scoring system for the clinical parameters (0 = not present; 1 = improving; 2 = stable; 3 = worsening or new). Due to their persistent nature, some items are only scored for their presence/absence at the moment of evaluation (e.g., strokes, cognitive impairment, malignancy), as indicated in Table 1. The four laboratory values (hemoglobin concentration, neutrophil count, lymphocyte count, and platelet count) are scored based on a reference table (supplementary Table S1), and the need for treatment escalation is scored categorically (yes = 2; no = 0). To take into account the different severity of some symptoms, or the patient-reported aspect, less weight is attributed to some items, as indicated in Table 1 (e.g., headache, mild livedo, arthralgia). The criteria used to select these minor items were: not life-threatening, not causing severe organ damage (as established by standard evaluation systems, such as Chronic Kidney Disease grades for kidney failure and Child Pugh scores for liver failure), or transient, with the exception of systemic symptoms of inflammation and of immunodeficiency/lymphoproliferation, considered to always reflect a severe clinical state. To differentiate between actual disease activity and the burden of chronic organ damage, we also propose a disease damage index, which includes 21 items representing the different organs and systems affected in DADA2. The scoring is weighted to differentiate between severe (2 points) and mild organ dysfunction (1 point), as indicated in Table 2. These scores should be calculated by a physician at a visit or retrospectively based on clinical records.

**Table 1:** The DADA2 disease activity index (DADA2AI). Items must be scored according to the following criteria: 0 = not present; 1 = improving; 2 = stable; 3 = worsening or new, except for the item “treatment escalation required” (yes = 2, no = 0) and for the laboratory values (see supplementary Table S1). Due to their persistent nature, some items are only scored for their presence/absence at the moment of evaluation (e.g., strokes, malignancy), as indicated in the scoring system (last column). Moreover, the score of aspecific and milder symptoms is divided by 2 to take into account lesser severity/self-reported aspects. Items must be scored according to symptoms/results at the moment of the evaluation (can also be applied retrospectively)

Category	Item	Description	Scoring system: 0 = not present; 1 = improving; 2 = stable; 3 = worsening or new onset
Constitutional	Fever	> 38 °C, infections excluded	0–1-2–3
	Anorexia, weight loss	(Unintentional)	0–1-2–3
	Hypertension		0–1-2–3
Neurological	Ischemic or hemorrhagic stroke		0–3
	TIA, focal neurological defects	Including convulsions	0–3
	Neuropathy, optic neuritis		0–1-2–3
	Other imaging findings	White matter changes, changes compatible with PRES, optic nerve atrophy, brain atrophy, leptomeningeal enhancement, vascular anomalies, other brain scan anomalies	0–3
	Aspecific symptoms	Headache, dizziness, amnesia, blurry vision (not caused by one of the events above)	0–1-2–3 and /2
Psychodevelopmental	Cognitive impairment	Isolated low IQ, learning disabilities	0–3
	Developmental delay	Motoric/cognitive	0–1-2–3 and /2
	Autism spectrum disorder		0–3 and /2
	ADHD		0–3 and /2
	Behavioral problems	(Not falling in one of the previous categories)	0–1-2–3 and /2
Skin	Vasculitis—severe	Severe livedo, severe chilblains, Raynaud, ulcers, digital infarcts, erythema nodosum, vasculitis	0–1-2–3
	Vasculitis—mild	Livedo, rash, chilblains	0–1-2–3 and /2
	Warts (verrucae), mollusca		0–1-2–3 and /2
	Aspecific rash, other	Urticaria, aphtosis, alopecia	0–1-2–3 and /2
Hepatic/gastrointestinal	Impaired liver function (mild), nodular regenerative hyperplasia	Elevation of transaminases > 3xULN, biopsy evidence of mild/moderate liver disease (hepatoportal sclerosis, hepatitis, portal vasculopathy, nodular regenerative hyperplasia), Child Pugh Score A	0–1-2–3 and /2
	Impaired liver function (moderate/severe), portal hypertension, liver failure	Presence of portal hypertension as estimated by MRI, ultrasound or measured by catheterization, varices as seen by endoscopy, Child Pugh Scores B and C	0–1-2–3
	GI inflammation	IBD, ileitis, appendicitis	0–1-2–3
	GI necrosis	Infarction, perforation	0–3
	Aspecific/other patient-reported	Abdominal pain	0–1-2–3 and /2
Musculoskeletal	Arthralgia		0–1-2–3 and /2
	Arthritis, myositis		0–1-2–3
	Myalgia		0–1-2–3 and /2
Nephrological	Proteinuria		0–1-2–3
	Kidney failure	CKD1-5	0–1-2–3
	TMA		0–1-2–3
Hematological and immunological	Hepatosplenomegaly	> 2SD as measured by ultrasound/MRI	0–1-2–3
	Lymphadenopathy		0–1-2–3
	Immune cytopenia or hemophagocytosis	AIHA, ITP, AI neutropenia, hemophagocytosis/HLH, TLGL	0–1-2–3
	Bone marrow failure/fibrosis	PRCA, BM fibrosis, BM dysplasia, increased CD8 + T cell infiltrate in BM	0–1-2–3
	Hypogammaglobulinemia	At least IgG below the reference for age	0–1-2–3
	Recurrent infections		0–1-2–3
	Herpes infections or viremia	HSV, VZV, EBV, CMV, HHV6	0–1-2–3
Other	Malignancy		0–3
	Uveitis, retinal vasculitis		0–1-2–3
Treatment escalation required	Yes/No		Yes: 2, no: 0
Laboratory values	Hb (no hemolysis, mg/dL)*		See supplementary Table S1 for scoring
	Hb (hemolysis proven, mg/dL)*		
	Neutrophils (n/µL)		
	Lymphocytes (n/µL)		
	Platelets (n*10^3^/µL)		

*ADHD* attention deficit and hyperactivity disorder, *AI* autoimmune, *AIHA* autoimmune hemolytic anemia, *BM* bone marrow, *CKD* chronic kidney disease, *CMV* cytomegalovirus, *EBV* Epstein-Barr virus, *HHV6* human herpesvirus 6, *HLH* hemophagocytic lymphohistiocytosis, *HSV* herpes simplex virus, *IBD* inflammatory bowel disease, *IQ* intelligence quotient, *ITP* immune thrombocytopenia, *PRCA* pure red cell aplasia, *PRES* posterior reversible encephalopathy syndrome, *TIA* transient ischemic attack, *TLGL* T large granular lymphocytes, *TMA* thrombotic microangiopathy, *ULN* upper limit of normal, *VZV* varicella-zoster virus

**Table 2:** The DADA2 disease damage index (DADA2DI). Items must be scored according to the following criteria: 0 = not present; 2 = present. The score of aspecific and milder symptoms is divided by 2 to take into account lesser severity. Items must be scored according to symptoms/results at the moment of the evaluation (can also be applied retrospectively)

Category	Item	Description	Scoring system: 0 = not present; 2 = present
Constitutional	Chronic arterial hypertension		0–2
Neurological	Ischemic stroke*		0–2
	Hemorrhagic stroke*		0–2
	Mononeuropathy		0–2
	Polyneuropathy		0–2
	Other imaging findings	White matter changes, changes compatible with PRES, optic nerve atrophy, brain atrophy, leptomeningeal enhancement, vascular anomalies, other brain scan anomalies	0–2
Psychodevelopmental	Cognitive impairment	Isolated low IQ, learning disabilities	0–2
	Developmental delay	Motoric/cognitive	0–2
	Autism spectrum disorder/ADHD		0–2
Skin	Digital infarcts or necrosis*		0–2
	Persistent alopecia		0–2 and /2
Hepatic/Gastrointestinal	Mild liver damage	Hepatoportal sclerosis, chronic hepatitis/chronically elevated AST-ALT without liver failure, portal vasculopathy, nodular regenerative hyperplasia, mild hepatic fibrosis, Child Pugh score A	0–2 and /2
	Severe liver damage	Presence of portal hypertension as estimated by MRI, ultrasound or measured by catheterization, varices as seen by endoscopy, moderate-severe hepatic fibrosis, Child Pugh Scores B and C	0–2
	GI necrosis*	GI resections, perforation	0–2
Musculoskeletal	Arthropathy with articular ankylosis/damage*		0–2
Nephrological	Kidney failure	CKD 1–2-3	0–2 and /2
	Kidney failure	CKD 4–5	0–2
	Kidney transplantation		0–2
Hematological and immunological	Bone marrow failure/fibrosis,	PRCA, BM fibrosis, BM dysplasia, increased CD8 + T cell infiltrate in BM	0–2
Other	Malignancy		0–2
	Uveitis, retinal vasculitis		0–2

^*^for these items, add 2 points for each subsequent separate episode of stroke/skin or GI necrosis/articular damage

*ADHD* attention deficit and hyperactivity disorder, *BM* bone marrow, *CKD* chronic kidney disease, *GI* gastrointestinal, *IQ* intelligence quotient, *MRI* magnetic resonance imaging, *PRCA* pure red cell aplasia, *PRES* posterior reversible encephalopathy syndrome

These severity indexes can be used to longitudinally monitor individual patients and assess disease burden and activity. In our opinion, the scores reflect the clinical status of the patients, as tested retrospectively and considering several time points in our cohort of 12 local DADA2 patients. The evaluation was conducted by two physicians separately, obtaining concurring results. We present the following two cases from clinical practice as an example (summarized in bullet form).

P1 is a 12-year-old boy who was diagnosed with DADA2 at the age of 3 years (previously described, supplementary E21,E22).

- Age 6 months: suspected transient erythroblastopenia of childhood triggered by human herpesvirus 6 (HHV6) infection
- Age 2 years: recurrent cutaneous herpes simplex infections, hepatosplenomegaly, lymphadenopathies, hypogammaglobulinemia, and inflammatory bowel disease (IBD) with elevated systemic inflammatory parameters. IBD was complicated by repeated obstructions requiring two partial ileal resections. He was started on immunosuppressive medication (steroids, sirolimus) and immunoglobulins
- Between the age of 3 and 5 years: recurrent transient ischemic attacks (TIAs), treated with prophylactic aspirin. DADA2 diagnosis. New episode of ileitis and obstruction, conservatively treated. Partial paresis of the third cranial nerve
- Five years of age: in the run-up to HSCT, he suffered a subarachnoid hemorrhagic stroke of the brainstem, characterized by headache and meningismus, and a flare of ileitis with appendicitis, treated with steroids, antibiotics, and etanercept. Ten of 10 matched unrelated HSCT after reduced intensity conditioning with alemtuzumab, treosulfan, and fludarabine
- Six years of age: post-HSCT course complicated by acute graft-versus-host-disease (GVHD) grade I of the skin, adenovirus and herpes zoster reactivation, and secondary loss of myeloid chimerism, fall of serum ADA2 enzyme activity levels, and onset of hemolytic anemia 8 months post-HSCT, not responsive to several lines of immune suppressants. He was treated with two hematopoietic stem cell boosts without conditioning, reaching again full myeloid chimerism and complete resolution of symptoms

We calculated the disease activity score at diagnosis (3 years old), just before HSCT (5 years old) and currently, after HSCT, obtaining scores of 15, 24.5, and 3.5, respectively (supplementary Table S2). The disease damage score is currently 4. These scores reflect the overall burden of disease as qualitatively evaluated by the physician.

P2 is a 13-year-old boy diagnosed with DADA2 at the age of 6 years.

- Age 4 months: persistent maculopapular skin rash and livedo and recurrent molluscum contagiosum
- Between 3 and 5 years: hypogammaglobulinemia with recurrent respiratory tract infections, for which immunoglobulin therapy was started
- Age 6 years: persistent fever, arthralgia, and vasculitis of the left lower leg with associated myositis, treated with non-steroidal anti-inflammatory drugs (NSAIDs). DADA2 diagnosis
- Age 7 years: two episodes of fevers and arthralgia, treated with NSAIDs, and then an episode of severe immune thrombocytopenia (ITP) and autoimmune hemolytic anemia (AIHA), treated with steroids followed by rituximab. He was started on etanercept with good clinical evolution
- Age 11 years: persistent fever and arthralgia. His treatment was switched to adalimumab, with resolution of symptoms. He was diagnosed with autism spectrum disorder aged 10 years

The disease activity index was calculated at diagnosis (6 years old), at 7 years old before and after start of therapy with etanercept, at 11 years old before switch to adalimumab, and at 13 years old, obtaining scores of 20.5, 23, 11, 16, and 8, respectively (supplementary Table S3). The disease damage score is currently 2.

The proposed DADA2 disease activity index (DADA2AI) and disease damage index (DADA2DI) contain parameters that reflect the entire clinical spectrum of DADA2, specifically addressing the most relevant features of inflammation, vasculitis, neurological involvement, immune deficiency, and bone marrow anomalies presented by the patients. They correspond to the main areas of disease and organ involvement identified by Fayand et al. in a large retrospective review of more than 300 patients and by Barron et al. in 60 patients (supplementary E19,E20). We have added a section on psychodevelopmental manifestations based on the personal and reported observation of increased prevalence of mild psychomotor delay and/or autism spectrum disorder/attention deficit and hyperactivity disorder among our patients (supplementary E23). The parameters have been divided into organ/system categories to avoid the ambiguity of general terms (e.g., “vasculitis” could refer to the brain, the skin, or the gut). The information is easily retrievable from a standard history and clinical examination performed by the physician during an outpatient visit, making the scores very practical. We made a distinction between disease activity and disease damage to be able to differentiate the burden of chronic, irreversible organ damage from the actual disease activity, which is potentially reversible and can reflect response to treatment. The use of both indexes allows the assessment of global disease severity.

The scoring system for DADA2AI takes into account the evolution of the symptoms in a patient and can be used both retrospectively and prospectively to assess intra- and inter-patient differences. It could be especially helpful as a measure of the natural evolution of the disease or of the response to a specific therapy in cohorts of patients. Moreover, the attribution of points based on the novelty, improvement, or worsening of a symptom removes in part the uncertainty and individual judgment intrinsic to other scoring systems, such as those requiring an attribution of severity. This has the downside of not differentiating between a milder and more severe presentation of the same symptom in different patients, thus probably reducing inter-patient differences. To mitigate this effect, the items have been carefully divided to differentiate between milder and more severe manifestations of the same type based on degree of organ damage, threaten to life, and reversibility (e.g., gastrointestinal inflammation and necrosis, severe and mild skin vasculitis, arthritis and arthralgia), attributing a half score to the milder presentations. Furthermore, this does not represent a problem when comparing intra-patient longitudinal data. The item “treatment escalation required” risks to be more subjective, but it reflects the physician’s and possibly the patient’s assessment of the overall evolution of the patient’s status. The validation of these scores would require on one hand their application to several hundreds or thousands of patients, to test their effectiveness in correctly estimating DADA2 disease activity and burden; on the other hand, they should be further validated by assessing the same real-patient exercises by different physicians to demonstrate the reproducibility of the evaluation; and finally, there would ideally be a positive correlation with a future highly specific and sensitive serum biomarker. These disease activity and disease damage indexes will be a good addition to general quality of life and performance indexes. Limitations of this study are the lack of extensive validation to confirm the reproducibility of the results among different physicians, a degree of uncertainty due to user-dependent differences in assessing the evolution of patients’ symptoms, and the fact that the results do not reflect the specific phenotype of the patients. As such, two patients with the same score could have completely different presentations. Of note, the different clinical items in the scores should individually all hint to a potential underlying diagnosis of DADA2, if no other valid diagnosis is made.

In conclusion, we propose a DADA2 disease activity index, DADA2AI, and disease damage index, DADA2DI, covering all clinical aspects of DADA2. We believe them to be a reliable representation of the clinical state of the patient, allowing straightforward longitudinal analyses of the disease burden, response to therapy, and disease activity and course in clinical and research setting. We welcome the use of these scores in prospective patient registry studies and drug clinical trials to learn more about the course of this disease.

### Supplementary Information

Below is the link to the electronic supplementary material.

Supplementary file1 (DOCX 35 KB)

Supplementary file2 (XLSX 15 KB)

## Data Availability

Not applicable.
