# Supplementary material for: Proposal for a Disease Activity Score and Disease Damage Score for ADA2 Deficiency: the DADA2AI and DADA2DI
Source: J Clin Immunol. 2023 Dec 22;44(1):25. doi: 10.1007/s10875-023-01638-w (PMC10739542; doi:10.1007/s10875-023-01638-w)
Supplement: Supplementary file 1 — Supplementary file1 (DOCX 35 KB) [file 10875_2023_1638_MOESM1_ESM.docx]

**Table S1.** Reference for the scoring of 5 laboratory values. *Either of but not both values of hemoglobin (Hb) must be scored, depending on the presence or not of hemolysis.

|  | **0** | **1** | **2** | **3** | **4** |
| --- | --- | --- | --- | --- | --- |
| Hb (no hemolysis, mg/dL)* | Normal | >11^§^ | 8-10.9 | <8 |  |
| Hb (hemolysis proven^#^, mg/dL)* | Normal |  | >10 | 8-9.9 | <8 |
| Neutrophils (n/µL) | Normal | >1500^§^ | 500-1500 | <500 |  |
| Lymphocytes (n/µL) | Normal | >1000^§^ | <1000 |  |  |
| Platelets (n*10^3^/µL) | Normal | >150^§^ | 50-150 | 20-50 | <20 |

^#^Proven by: positive direct Coombs test, increased LDH, increased bilirubin; ^§^below age-specific reference

**Table S2.** Example of retrospective application of the DADA2 disease severity score to a patient (P1) with DADA2 at 3 timepoints (at diagnosis, before HSCT and after HSCT). HSCT was performed 1.5 months after the date of the pre-HSCT score.

| **Category** | **Item** | **At diagnosis**  **(3 y.o.)** | **Pre-HSCT**  **(5 y.o.)** | **Post-HSCT**  **(12 y.o.)** |
| --- | --- | --- | --- | --- |
| **Constitutional** | Fever | 0 | 0 | 0 |
|  | Anorexia, weight loss | 0 | 0 | 0 |
|  | Hypertension | 0 | 3 | 0 |
| **Neurological** | New ischemic or hemorrhagic stroke | 0 | 3 | 0 |
|  | TIA, focal neurological defects | 3 | 0 | 0 |
|  | Neuropathy, optic neuritis | 0 | 0 | 0 |
|  | Other imaging findings | 0 | 0 | 0 |
|  | Aspecific symptoms | 0 | 2/2 | 0 |
| **Psychodevelopmental** | Cognitive impairment | 0 | 0 | 0 |
|  | Developmental delay | 0 | 0 | 0 |
|  | Autism spectrum disorder | 0 | 0 | 0 |
|  | ADHD | 0 | 0 | 0 |
|  | Behavioral problems | 0 | 0 | 3/2 |
| **Skin** | Vasculitis - severe | 0 | 0 | 0 |
|  | Vasculitis - mild | 0 | 0 | 0 |
|  | Warts (verrucae), mollusca | 0 | 0 | 0 |
|  | Aspecific rash, other | 0 | 3 | 0 |
| **Hepatic/Gastrointestinal** | Impaired liver function (mild), nodular regenerative hyperplasia | 0 | 2 | 0 |
|  | Impaired liver function (moderate/severe), portal hypertension, liver failure | 0 | 0 | 0 |
|  | GI inflammation | 2 | 3 | 0 |
|  | GI necrosis | 0 | 0 | 0 |
|  | Aspecific/other patient reported | 0 | 0 | 2/2 |
| **Musculoskeletal** | Arthralgia | 0 | 1/2 | 0 |
|  | Arthritis or myositis | 0 | 0 | 0 |
|  | Myalgia | 0 | 0 | 0 |
| **Nephrological** | Proteinuria | 0 | 0 | 0 |
|  | Kidney failure | 0 | 0 | 0 |
|  | Thrombotic microangiopathy (TMA) | 0 | 0 | 0 |
| **Hematological and immunological** | Hepatosplenomegaly | 0 | 2 | 0 |
|  | Lymphadenopathy | 2 | 0 | 0 |
|  | Immune cytopenia or hemophagocytosis | 0 | 0 | 0 |
|  | Bone marrow failure/fibrosis | 0 | 0 | 0 |
|  | Hypogammaglobulinemia | 2 | 2 | 0 |
|  | Recurrent infections or viremia | 0 | 0 | 0 |
|  | Herpes infections or viremia | 0 | 0 | 0 |
| **Other** | Malignancy | 0 | 0 | 0 |
|  | Uveitis, retinal vasculitis | 0 | 0 | 0 |
| **Treatment escalation required** | Yes / No | 2 | 2 | 0 |
| **Laboratory values** | Hb (no hemolysis, mg/dL)* | 2 | 2 | 1 |
|  | Hb (hemolysis proven, mg/dL)* | / | / | / |
|  | Neutrophils (n/µL) | 2 | 0 | 0 |
|  | Lymphocytes (n/µL) | 0 | 1 | 0 |
|  | Platelets (n*10^3^/µL) | 0 | 0 | 0 |
| **Total** |  | **15** | **24.5** | **3.5** |

**Table S3.** Example of retrospective application of the DADA2 disease severity score to a patient (P2) with DADA2 at 5 timepoints (at diagnosis, before and after start of etanercept, before and after start of adalimumab).

| **Category** | **Item** | **At diagnosis**  **(6 y.o.)** | **Pre-etanercept**  **(7 y.o.)** | **Post-etanercept**  **(7 y.o.)** | **Pre-adalimumab (11 y.o.)** | **Post- adalimumab (13 y.o.)** |
| --- | --- | --- | --- | --- | --- | --- |
| **Constitutional** | Fever | 3 | 2 | 1 | 3 | 0 |
|  | Anorexia, weight loss | 2 | 0 | 0 | 0 | 0 |
|  | Hypertension | 0 | 0 | 0 | 0 | 0 |
| **Neurological** | New ischemic or hemorrhagic stroke | 0 | 0 | 0 | 0 | 0 |
|  | TIA, focal neurological defects | 0 | 0 | 0 | 0 | 0 |
|  | Neuropathy, optic neuritis | 0 | 0 | 0 | 0 | 0 |
|  | Other imaging findings | 0 | 0 | 0 | 0 | 0 |
|  | Aspecific symptoms | 0 | 0 | 0 | 3/2 | 2/2 |
| **Psychodevelopmental** | Cognitive impairment | 0 | 0 | 0 | 0 | 0 |
|  | Developmental delay | 0 | 0 | 0 | 0 | 0 |
|  | Autism spectrum disorder | 0 | 0 | 0 | 2 | 2 |
|  | ADHD | 0 | 0 | 0 | 0 | 0 |
|  | Behavioral problems | 0 | 0 | 0 | 0 | 0 |
| **Skin** | Vasculitis - severe | 0 | 0 | 0 | 0 | 0 |
|  | Vasculitis - mild | 2/2 | 0 | 0 | 0 | 0 |
|  | Warts (verrucae), mollusca | 2/2 | 3/2 | 2/2 | 0 | 0 |
|  | Aspecific rash, other | 0 | 0 | 0 | 0 | 0 |
| **Hepatic/Gastrointestinal** | Impaired liver function (mild), nodular regenerative hyperplasia | 0 | 0 | 0 | 0 | 0 |
|  | Impaired liver function (moderate/severe), portal hypertension, liver failure | 0 | 0 | 0 | 0 | 0 |
|  | GI inflammation | 0 | 0 | 0 | 0 | 0 |
|  | GI necrosis | 0 | 0 | 0 | 0 | 0 |
|  | Aspecific/other patient reported | 0 | 0 | 0 | 0 | 0 |
| **Musculoskeletal** | Arthralgia | 3/2 | 3/2 | 0 | 3/2 | 0 |
|  | Arthritis or myositis | 3 | 0 | 0 | 0 | 0 |
|  | Myalgia | 0 | 0 | 0 | 0 | 0 |
| **Nephrological** | Proteinuria | 0 | 0 | 0 | 0 | 0 |
|  | Kidney failure | 0 | 0 | 0 | 0 | 0 |
|  | Thrombotic microangiopathy (TMA) | 0 | 0 | 0 | 0 | 0 |
| **Hematological and immunological** | Hepatosplenomegaly | 0 | 3 | 2 | 0 | 0 |
|  | Lymphadenopathy | 3 | 0 | 0 | 0 | 0 |
|  | Immune cytopenia or hemophagocytosis | 0 | 3 | 0 | 0 | 0 |
|  | Bone marrow failure/fibrosis | 0 | 0 | 0 | 0 | 0 |
|  | Hypogammaglobulinemia | 2 | 2 | 2 | 2 | 2 |
|  | Recurrent infections or viremia | 0 | 0 | 0 | 3 | 0 |
|  | Herpes infections or viremia | 0 | 0 | 0 | 0 | 0 |
| **Other** | Malignancy | 0 | 0 | 0 | 0 | 0 |
|  | Uveitis, retinal vasculitis | 0 | 0 | 0 | 0 | 0 |
| **Treatment escalation required** | Yes / No | 2 | 2 | 0 | 2 | 0 |
| **Laboratory values** | Hb (no hemolysis, mg/dL)* | 2 | / | 2 | 1 | 1 |
|  | Hb (hemolysis proven, mg/dL)* | / | 4 | 0 | / | / |
|  | Neutrophils (n/µL) | 0 | 0 | 2 | 1 | 2 |
|  | Lymphocytes (n/µL) | 0 | 0 | 1 | 1 | 0 |
|  | Platelets (n*10^3^/µL) | 0 | 4 | 0 | 0 | 0 |
| **Total** |  | **20.5** | **23** | **11** | **16** | **8** |

Supplemental bibliography

E1. Hashem H, Kelly SJ, Ganson NJ, Hershfield MS. Deficiency of Adenosine Deaminase 2 (DADA2), an Inherited Cause of Polyarteritis Nodosa and a Mimic of Other Systemic Rheumatologic Disorders. Curr Rheumatol Rep. 2017 Oct 5;19(11):70.

E2. Lee PY, Kellner ES, Huang Y, Furutani E, Huang Z, Bainter W, et al. Genotype and functional correlates of disease phenotype in deficiency of adenosine deaminase 2 (DADA2). Journal of Allergy and Clinical Immunology. 2020 Jun 1;145(6):1664-1672.e10.

E3. Cipe FE, Aydogmus C, Serwas NK, Keskindemirci G, Boztuğ K. Novel Mutation in CECR1 Leads to Deficiency of ADA2 with Associated Neutropenia. J Clin Immunol. 2018 Apr 1;38(3):273–7.

E4. Alsultan A, Basher E, Alqanatish J, Mohammed R, Alfadhel M. Deficiency of ADA2 mimicking autoimmune lymphoproliferative syndrome in the absence of livedo reticularis and vasculitis. Pediatric Blood & Cancer. 2018;65(4):e26912.

E5. Schepp J, Bulashevska A, Mannhardt-Laakmann W, Cao H, Yang F, Seidl M, et al. Deficiency of Adenosine Deaminase 2 Causes Antibody Deficiency. J Clin Immunol. 2016 Apr 1;36(3):179–86.

E6. Trotta L, Martelius T, Siitonen T, Hautala T, Hämäläinen S, Juntti H, et al. ADA2 deficiency: Clonal lymphoproliferation in a subset of patients. Journal of Allergy and Clinical Immunology. 2018 Apr 1;141(4):1534-1537.e8.

E7. Michniacki TF, Hannibal M, Ross CW, Frame DG, DuVall AS, Khoriaty R, et al. Hematologic Manifestations of Deficiency of Adenosine Deaminase 2 (DADA2) and Response to Tumor Necrosis Factor Inhibition in DADA2-Associated Bone Marrow Failure. J Clin Immunol. 2018 Feb 1;38(2):166–73.

E8. Andriessen MVE, Legger GE, Bredius RGM, van Gijn ME, Hak AE, Muller PCEH, et al. Clinical Symptoms, Laboratory Parameters and Long-Term Follow-up in a National DADA2 Cohort. J Clin Immunol. 2023 Jun 5.

E9. Hashem H, Kumar AR, Müller I, Babor F, Bredius R, Dalal J, et al. Hematopoietic stem cell transplantation rescues the hematological, immunological, and vascular phenotype in DADA2. Blood. 2017 Dec 14;130(24):2682–8.

E10. Hashem H, Bucciol G, Ozen S, Unal S, Bozkaya IO, Akarsu N, et al. Hematopoietic Cell Transplantation Cures Adenosine Deaminase 2 Deficiency: Report on 30 Patients. J Clin Immunol. 2021 Oct 1;41(7):1633–47.

E11. England BR, Tiong BK, Bergman MJ, Curtis JR, Kazi S, Mikuls TR, et al. 2019 Update of the American College of Rheumatology Recommended Rheumatoid Arthritis Disease Activity Measures. Arthritis Care & Research. 2019;71(12):1540–55.

E12. Swart JF, Dijkhuizen EHP van, Wulffraat NM, Roock S de. Clinical Juvenile Arthritis Disease Activity Score proves to be a useful tool in treat-to-target therapy in juvenile idiopathic arthritis. Annals of the Rheumatic Diseases. 2018 Mar 1;77(3):336–42.

E13. Price-Kuehne FE, Eleftheriou D, Ozen S, Beresford M, Dolezalova P, Brogan PA. Preliminary validation of the paediatric vasculitis activity score (PVAS). Pediatric Rheumatology. 2011 Sep 14;9(1):O15.

E14. Piram M, Koné-Paut I, Lachmann HJ, Frenkel J, Ozen S, Kuemmerle-Deschner J, et al. Validation of the Auto-Inflammatory Diseases Activity Index (AIDAI) for hereditary recurrent fever syndromes. Annals of the Rheumatic Diseases. 2014 Dec 1;73(12):2168–73.

E15. Romero-Diaz J, Isenberg D, Ramsey-Goldman R. Measures of adult systemic lupus erythematosus: Updated Version of British Isles Lupus Assessment Group (BILAG 2004), European Consensus Lupus Activity Measurements (ECLAM), Systemic Lupus Activity Measure, Revised (SLAM-R), Systemic Lupus Activity Questionnaire for Population Studies (SLAQ), Systemic Lupus Erythematosus Disease Activity Index 2000 (SLEDAI-2K), and Systemic Lupus International Collaborating Clinics/American College of Rheumatology Damage Index (SDI). Arthritis Care & Research. 2011;63(S11):S37–46.

E16. Ameratunga R. Assessing Disease Severity in Common Variable Immunodeficiency Disorders (CVID) and CVID-Like Disorders. Frontiers in Immunology. 2018 Sep 28;9(2130).

E17. Tesch VK, Abolhassani H, Shadur B, Zobel J, Mareika Y, Sharapova S, et al. Long-term outcome of LRBA deficiency in 76 patients after various treatment modalities as evaluated by the immune deficiency and dysregulation activity (IDDA) score. Journal of Allergy and Clinical Immunology. 2020 May 1;145(5):1452–63.

E18. Seidel MG, Tesch VK, Yang L, Hauck F, Horn AL, Smolle MA, et al. The Immune Deficiency and Dysregulation Activity (IDDA2.1 ‘Kaleidoscope’) Score and Other Clinical Measures in Inborn Errors of Immunity. J Clin Immunol. 2022 Apr 1;42(3):484–98.

E19. Barron KS, Aksentijevich I, Deuitch NT, Stone DL, Hoffmann P, Videgar-Laird R, et al. The Spectrum of the Deficiency of Adenosine Deaminase 2: An Observational Analysis of a 60 Patient Cohort. Front Immunol. 2022 Jan 10:12:811473.

E20. Fayand A, Chasset F, Boutboul D, Queyrel V, Tieulié N, Guichard I, et al. DADA2 diagnosed in adulthood versus childhood: A comparative study on 306 patients including a systematic literature review and 12 French cases. Seminars in Arthritis and Rheumatism. 2021 Dec 1;51(6):1170–9.

E21. Van Eyck L, Hershfield MS, Pombal D, Kelly SJ, Ganson NJ, Moens L, et al. Hematopoietic stem cell transplantation rescues the immunologic phenotype and prevents vasculopathy in patients with adenosine deaminase 2 deficiency. Journal of Allergy and Clinical Immunology. 2015 Jan 1;135(1):283-287.e5.

E22. Bucciol G, Delafontaine S, Segers H, Bossuyt X, Hershfield MS, Moens L, et al. Hematopoietic Stem Cell Transplantation in ADA2 Deficiency: Early Restoration of ADA2 Enzyme Activity and Disease Relapse upon Drop of Donor Chimerism. J Clin Immunol. 2017 Nov 1;37(8):746–50.

E23. Dzhus M, Ehlers L, Wouters M, Jansen K, Schrijvers R, De Somer L, et al. A Narrative Review of the Neurological Manifestations of Human Adenosine Deaminase 2 Deficiency. J Clin Immunol. 2023 Aug 7.
